# Two new species of the genus *Hydropsyche* Pictet, 1834 (Trichoptera, Hydropsychidae) from the Middle East and Caucasus ecoregions

**DOI:** 10.3897/BDJ.14.e191076

**Published:** 2026-05-12

**Authors:** Halil Ibrahimi, Dora Hlebec

**Affiliations:** 1 Department of Biology, Faculty of Mathematics and Natural Sciences, University of Prishtina, Prishtina, Kosovo Department of Biology, Faculty of Mathematics and Natural Sciences, University of Prishtina Prishtina Kosovo; 2 Department of Biology, Faculty of Science, University of Zagreb, Zagreb, Croatia Department of Biology, Faculty of Science, University of Zagreb Zagreb Croatia

**Keywords:** Azerbaijan, Iran, Türkiye, caddisfly diversity, species description, *Hydropsyche angustipennis* species group

## Abstract

**Background:**

The knowledge about the genus *Hydropsyche* Pictet, 1834 in the Middle East and Caucasus ecoregions is still scarce with several species described during the past years.

**New information:**

In this paper, we describe two new species within the *Hydropsyche guttata* species cluster from several localities in the Middle East and Caucasus ecoregions. *Hydropsyche hindrajab* sp. nov. is reported from Azerbaijan, Iran and Türkiye, while *Hydropsyche fitesa* sp. nov. is reported from Iran. Males of these newly-discovered species are compared with their closest congeners: *Hydropsyche iranica* Malicky, 1977, *H. sciligra* Malicky, 1977 and *H. tigrata* Malicky, 1974. For *Hydropsyche hindrajab* sp. nov., we applied an integrative taxonomic approach combining morphological examination and molecular data, while *Hydropsyche fitesa* sp. nov. is described, based on morphological characters only. To elucidate phylogenetic and taxonomic relationships and to corroborate the distinctiveness of *Hydropsyche hindrajab* sp. nov., we analysed mitochondrial cytochrome *c* oxidase subunit I (*COI*) sequences and applied three species delimitation methods (ASAP, ABGD, bPTP). Although the two phylogenetic approaches yielded somewhat different topologies, all species delimitation analyses supported the recognition of *Hydropsyche hindrajab* sp. nov. as a distinct evolutionary lineage.

This study contributes to the knowledge of the *Hydropsyche* fauna of the Middle East and Caucasus ecoregions and demonstrates the utility of integrative taxonomy in resolving species boundaries within morphologically close species complexes.

## Introduction

The caddisfly faunas of Azerbaijan, Iran and Türkiye are documented to differing degrees, yet substantial knowledge gaps persist in all three countries. In Iran, many regions lack comprehensive surveys on the presence and distribution of this diverse order of aquatic insects. In recent years, several studies have significantly contributed to our understanding of Iranian caddisflies, including the family Hydropsychidae, by describing new species and recording previously undocumented occurrences (e.g. Mey (2004), Ibrahimi et al. (2024a)). Knowledge of the Hydropsychidae in Türkiye is comparatively better established than in Iran and Azerbaijan, having expanded substantially over recent decades, with several endemic species (e.g. Sipahiler and Malicky (1987), Sipahiler (2004), Sipahiler (2010), Sipahiler (2016)). In contrast, knowledge of Hydropsychidae in Azerbaijan, similar to the broader state of caddisfly research in general, remains incomplete, despite increased progress in recent years (Oláh et al. 2020, Oláh and Vinçon 2024, Oláh et al. 2025).

The *Hydropsyche guttata* species cluster, belonging to the *H. angustipennis* species group, comprises species distributed across the Mediterranean Region and adjacent parts of Europe, North Africa and western Asia (Kumanski and Botosaneanu 1974, Malicky 1977, Oláh and Johanson 2008). Species of this cluster are delimited primarily by morphological characters of male genitalia (Mosely 1939, Kumanski and Botosaneanu 1974, Malicky 1977), characterised by fused, strongly sclerotised endothecal processes and the absence of digitiform ventroapical setose lobes, as well as angular subapical lateral projections on the phallotheca (Malicky 1977). Based on these morphology-based species hypotheses, molecular data provide an independent, complementary line of evidence to evaluate species limits and refine species boundaries; therefore, an integrative approach combining morphology with DNA data would be effective for delimiting species and assessing diversity within the cluster.

In this paper, we describe two new species of the genus *Hydropsyche* from the Middle East and Caucasus ecoregions, including molecular analysis for one of these species.

## Materials and methods

### Study area

Type localities of two new species are located in Iran, while paratypes were collected beside Iran, in Azerbaijan and Türkiye (Table 1). Fig. 4 was created, based on a base map adapted from *Outline map of Middle East (cropped).svg*/*Outline map of Middle East.svg*, Wikimedia Commons, CC BY-SA.

Type locality of *Hydropsyche fitesa* sp. nov. is located below the Shalmash Falls consisting of three waterfalls, with the southernmost being the tallest and most prominent. The Chamyaman River is a tributary of the Little Zab River, both originating in the Zagros Mountains (Ghaderi et al. 2023). The substrate consisted predominantly of stones, pebbles, gravel and fine sediment, with sparse riparian vegetation.

Type locality of *Hydropsyche hindrajab* sp. nov. is situated along the Bardehsur River near Bardehsur Village (Urmia County, West Azerbaijan Province, Iran). The substrate consisted of stones, pebbles, gravel and fine sediment, with sparse riparian vegetation along the banks.

### Specimen collection and morphological examination

Adult caddisflies were collected using entomological nets and UV light traps and preserved in 80% ethanol. The abdomens were cleared in potassium hydroxide (KOH) and are stored in glycerine, while the remaining specimens are preserved in 80% ethanol. Specimens are deposited in the collection of the Faculty of Mathematics and Natural Sciences, University of Prishtina, Prishtina, Kosovo, under the collection ‘Iran’ (HIFMNSUPM).

### DNA extraction, PCR amplification and sequencing

For *Hydropsyche hindrajab* sp. nov., tissue samples (two legs) were taken from two specimens for molecular analysis. Total genomic DNA was extracted using the E.Z.N.A. Tissue DNA Kit (Omega Bio-tek, Georgia, USA) following the manufacturer’s protocol. The standard 658-bp barcode region of the mitochondrial cytochrome *c* oxidase subunit I (*COI*) gene was amplified using universal primers LCO-1490/HCO-2198 (Folmer et al. 1994) under the following PCR conditions: initial denaturation at 95°C for 2 min; followed by 35 cycles of denaturation at 95°C for 30 s, annealing at 50°C for 30 s and extension at 72°C for 1 min; with a final extension step at 72°C for 10 min. PCR amplifications were performed in 20 μl reaction mixtures containing 1 x DreamTaq™ reaction buffer, 0.2 mM dNTP mix, 0.5 μM of each primer, 1.0 U of DreamTaq™ DNA Polymerase (Thermo Fisher Scientific Inc., USA) and 1 μl of DNA eluate. PCR products were purified and bidirectionally sequenced by Macrogen Inc. (Amsterdam, The Netherlands) using the amplification primers. The sequences obtained in this study have been deposited in the BOLD Systems Database Portal (HYHIN001-26 and HYHIN002-26) and in GenBank under the accession numbers PZ303390 and PZ303391. The description of *Hydropsyche fitesa* sp. nov. is based on male morphology only, as attempts to obtain *COI* sequences from the available specimens were unsuccessful.

### Sequence analysis, phylogenetic analyses and species delimitation

**Figure 1.**
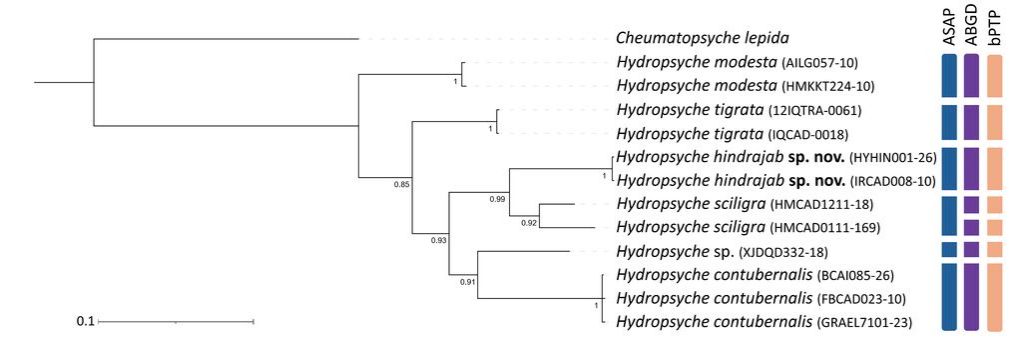
Bayesian Inference (BI) phylogeny, based on *COI* sequences of *Hydropsyche* species, rooted with *Cheumatopsyche lepida*. Coloured bars on the right indicate species delimitation results from ASAP, ABGD and bPTP analyses. Node support values represent Bayesian posterior probabilities (PP). Sequence details and BOLD Process IDs are provided in Table 1.

Manual editing and visual inspection of sequence chromatograms were performed in Geneious Prime 2025.0.3 (Biomatters, Auckland, New Zealand). Quality assessment procedures included examining chromatograms for the presence of double peaks and evaluating amino acid translations to check for the presence of stop codons. The final dataset of 18 sequences included two newly-obtained sequences from *Hydropsyche hindrajab* sp. nov. and 16 additional sequences retrieved from BOLD Systems Database Portal (Table 1). The "auto" alignment strategy in MAFFT v. 7 (Katoh et al. 2019) was employed for sequence alignment. Sequences were collapsed into haplotypes using FaBox v. 1.61 (Villesen 2007), resulting in 13 unique haplotypes. *Cheumatopsyche lepida* (Pictet, 1834) (GBEPT2117-15) was used as the outgroup. Two phylogenetic approaches were applied using a 658 bp long alignment that was codon-partitioned: Bayesian Inference (BI) using MrBayes v. 3.2.7 (Ronquist et al. 2012) (Fig. 1) and Maximum Likelihood (ML) using IQ-TREE v. 2.0.3 (Minh et al. 2020) with 5,000 ultrafast bootstrap replicates (Hoang et al. 2018) (Suppl. material 1). ModelFinder (Kalyaanamoorthy et al. 2017), implemented in IQ-TREE, determined the optimal substitution model for each partition (GTR+G). In the BI analysis, two independent MCMC runs were conducted with four chains each for 50 million generations, sampling every 2,000 generations. Parameters were evaluated for convergence and stationarity using Tracer v. 1.7.1 (Rambaut et al. 2018), with 25% of trees removed as burn-in. All analyses were executed on the CIPRES Science Gateway (Miller et al. 2010).

Uncorrected pairwise genetic distances (*p*-distances) were calculated in MEGA-X v. 10.2.6 (Kumar et al. 2018) using the pairwise deletion option. Three complementary methods were applied to delimit species boundaries. The ASAP method (Puillandre et al. 2021) identified molecular operational taxonomic units (MOTUs) using *p*-distance calculations under default programme settings. ABGD (Puillandre et al. 2012) employed Kimura 2-parameter distances with prior intraspecific divergence limits set at Pmin = 0.005 and Pmax = 0.1 and a relative gap width (X) of 1. The Bayesian Poisson Tree Processes (bPTP) approach (Zhang et al. 2013) utilised the IQ-TREE Maximum Likelihood phylogenetic tree as input, with MCMC analyses conducted for 500,000 generations. A thinning interval of 200 and burn-in of 20% were applied and species delimitations were based on the partition receiving the highest Bayesian support.

## Taxon treatments

### Hydropsyche hindrajab

Ibrahimi sp. nov.

5D84D590-D458-5919-A8A0-5ED798AD35E9

3B7FF3B6-B5F6-4637-9114-B9B616E3D86F

#### Materials

- **Type status:** Holotype. **Occurrence:** recordedBy: Halil Ibrahimi; individualCount: 1; sex: male; lifeStage: adult; occurrenceID: 59D53473-BB82-5F32-9296-A2F5BBEC7628; **Location:** continent: Asia; country: Iran; stateProvince: West Azerbaijan; county: Urmia; locality: Bardehsur Village, Bardehsur River; decimalLatitude: 37.43728; decimalLongitude: 44.82747; **Identification:** identifiedBy: Halil Ibrahimi; **Event:** samplingProtocol: entomological net; year: 2021; month: 7; day: 21
- **Type status:** Paratype. **Occurrence:** recordedBy: Halil Ibrahimi; individualCount: 3; sex: male; lifeStage: adult; occurrenceID: F2DC02B3-3517-5FF6-8A3E-4328650CF6E9; **Location:** continent: Asia; country: Iran; stateProvince: West Azerbaijan; county: Urmia; locality: Bardehsur Village, Bardehsur River; decimalLatitude: 37.43728; decimalLongitude: 44.82747; **Identification:** identifiedBy: Halil Ibrahimi; **Event:** samplingProtocol: entomological net; year: 2021; month: 7; day: 21
- **Type status:** Paratype. **Occurrence:** recordedBy: A. Mirzayev; individualCount: 3; sex: male; lifeStage: adult; occurrenceID: A2FFDFA7-56A4-55A4-90A8-5C1DA0B86EA7; **Location:** continent: Asia; country: Azerbaijan; stateProvince: Nakchivan Autonomous Republic; locality: Shikhmahmud, Nakchivanchay River; decimalLatitude: 39.245903; decimalLongitude: 45.443227; **Identification:** identifiedBy: Halil Ibrahimi; **Event:** samplingProtocol: entomological net; year: 2023; month: 6; day: 21
- **Type status:** Paratype. **Occurrence:** recordedBy: A. Mirzayev; individualCount: 1; sex: male; lifeStage: adult; occurrenceID: C5E3AB8F-0D10-51B1-B6FA-490FB1DE3873; **Location:** continent: Asia; country: Türkiye; municipality: Caldoran; locality: Sarigol Stream; decimalLatitude: 39.147397; decimalLongitude: 43.956664; **Identification:** identifiedBy: Halil Ibrahimi; **Event:** samplingProtocol: entomological net; year: 2023; month: 6; day: 12

#### Description

**Figure 2.**
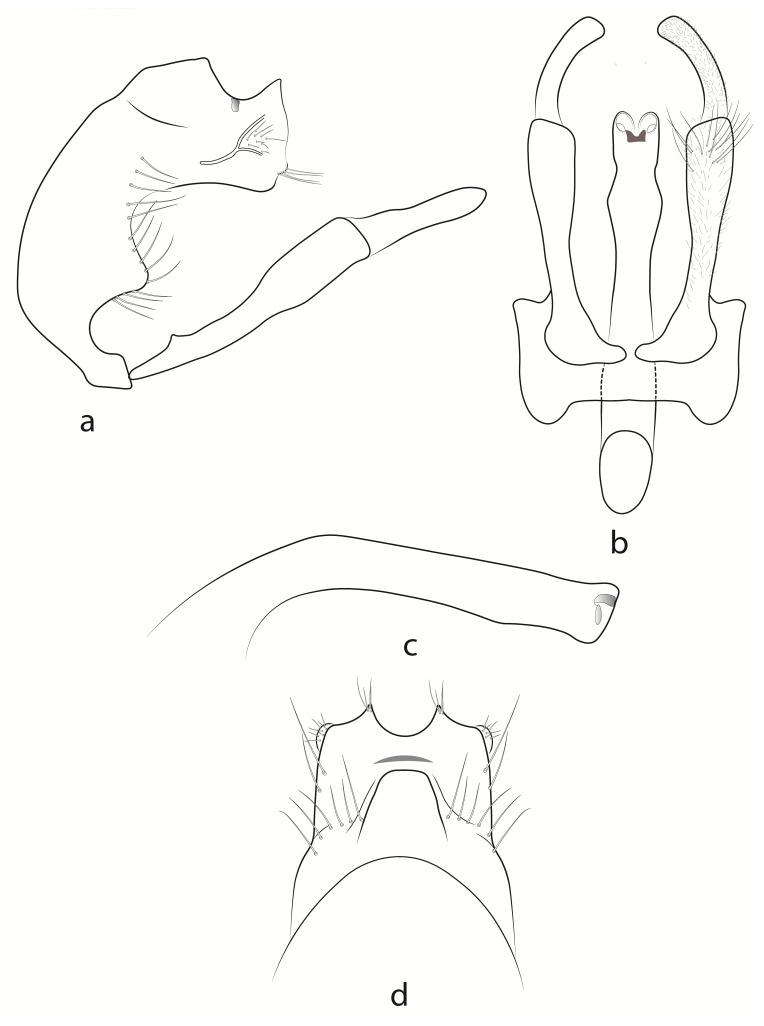
Male genitalia of *Hydropsyche hindrajab* sp. nov.: **a** lateral view; **b** ventral view; **c** phallus, lateral view; **d** dorsal view.

**Male** (Fig. 2). Fore-wing length 7.5 mm, hind-wing length 5.6 mm, uniformly pale castaneous. Antennae and palps dark brown, head and thorax dorsally black, first and second pairs of legs brown, tibia and femur of third pair dark brown, tarsal segments brown.

Abdominal segment IX with dorsal median keel; segment approximately three times as tall as its greatest length, anterior margin convex; apical lobe on posterolateral margin large, roughly triangular. Median keel in dorsal view roughly rectangular with straight to rounded apical margin and wide base. Intersegmental profile between segments IX and X wide, deep in lateral view. Segment X roughly quadrate, with medium-high, pointed dorsoapical lobes in dorsal view and wide, U-shaped excision in between.

Inferior appendages longer than apex of segment X, each with clavate coxopodite in lateral view, bearing few elongate setae distally and shorter ones throughout. In lateral view, coxopodites approximately twice as long as the harpago; harpago flattened, slightly wider basally and pointed apically.

Phallus in lateral view wide and strongly curved basally, narrowing along remaining length with slightly wider, indented apex; in ventral view, with rounded, moderately broad pre-apical swelling, followed by short constriction and secondary apical swelling with nearly straight lateral margins, apex subtruncate, with broad mesal emargination, forming two rounded apicolateral lobes.

##### Remarks

Fore-wing length 7.5–8.1 mm, hind-wing length 5.5–6.0 mm in paratypes.

#### Diagnosis

Males of *Hydropsyche hindrajab* sp. nov. are closest to those of *Hydropsyche tigrata* and *Hydropsyche sciligra*, but they differ in several aspects of habitus and genitalia. Firstly, *Hydropsyche hindjarab* sp. nov. has uniformly pale, chestnut-coloured fore-wings, in contrast to its closest congeners, which have yellow to brown fore-wings speckled with dark brown patches. Additionally, *Hydropsyche hindrajab* sp. nov. possesses two pairs of apical swellings on the phallus, with parallel margins on the apical swellings in ventral view, whereas in *H. sciligra*, the margins of the apical swellings are divergent. *Hydropsyche tigrata*, on the other hand, has only one pair of subapical swellings, which are markedly more pronounced than in the other two species, in both lateral and ventral views.

The dorsal keel of segment IX in *H. hindrajab* sp. nov. is large, roughly rectangular, with a long base and a shorter, nearly rounded apex in dorsal view. In contrast, the dorsal keels of *H. sciligra* and *H. tigrata* are considerably smaller and trapezoidal in shape. The shape of the harpago differs distinctly across the three species in ventral view: it is bluntly truncated in *H. tigrata*, squarely truncated in *H. hindrajab* sp. nov. and somewhat rounded with a ventroapically pointed tip in *H. sciligra*. The apices of segment X in *H. hindrajab* sp. nov. are low and pointed, almost rounded in *H. sciligra* and very high and pointed in *H. tigrata*.

#### Etymology

The species is dedicated to Hind Rajab, a five-year-old Palestinian girl killed by Israeli forces in 2024 in Gaza.

### Hydropsyche fitesa

Ibrahimi sp. nov.

1B81964D-84AD-5001-A81E-39E6694305AD

6C52E371-E60C-4C01-B4AA-95F3D15745EB

#### Materials

- **Type status:** Holotype. **Occurrence:** recordedBy: H. Ibrahimi; individualCount: 1; sex: male; lifeStage: adult; occurrenceID: 3AF2A177-2AD8-5889-AD20-5ED40A88FE4D; **Location:** continent: Asia; country: Iran; county: Sardasht; locality: Chamyaman River, Shalmash Waterfalls; decimalLatitude: 36.09779; decimalLongitude: 45.49293; **Event:** samplingProtocol: UV light trap; year: 2021; month: 7; day: 28
- **Type status:** Paratype. **Occurrence:** recordedBy: H. Ibrahimi; individualCount: 1; sex: male; lifeStage: adult; occurrenceID: B4D17129-F400-56CF-A31F-B1319D693E5E; **Location:** continent: Asia; country: Iran; county: Sardasht; locality: Chamyaman River, Shalmash Waterfalls; decimalLatitude: 36.09779; decimalLongitude: 45.49293; **Event:** samplingProtocol: UV light trap; year: 2021; month: 7; day: 28

#### Description

**Figure 3.**
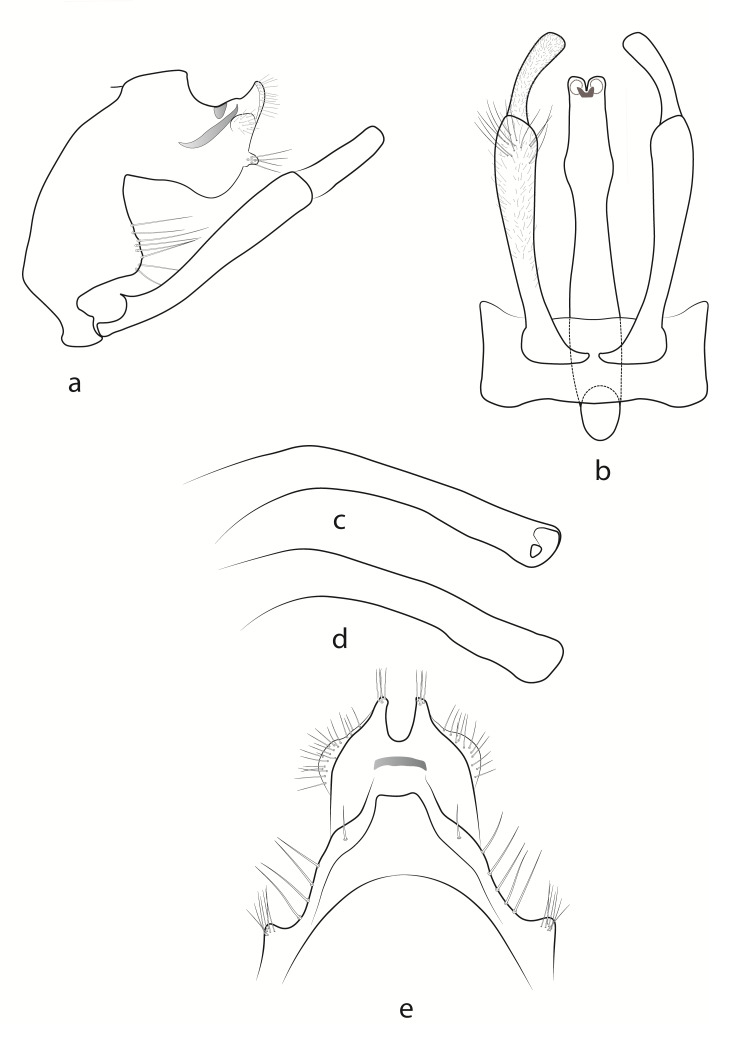
Male genitalia of *Hydropsyche fitesa* sp. nov.: **a** lateral view; **b** ventral view; **c** phallus, lateral view; **d** variation of phallus, lateral view; **e** dorsal view.

**Male** (Fig. 3). Fore-wing length 8.1 mm, light brown, irregularly speckled with darker patches; hind-wing length 6 mm, light brown. Antennae and palps dark brown, head and thorax dorsally dark brown and the first and second pairs of legs brown. Tibia and femur of the third pair dark brown, tarsal segments brown.

Abdominal segment IX with dorsal median keel, segment about three times as tall as its greatest length; anterior margin convex, slightly incised basally, apical lobe on posterolateral margin large, roughly triangular. Median keel in dorsal view roughly rectangular, with straight apical margin and wide base. Intersegmental profile between segments IX and X in lateral view wide and deep, subdistally stepped. Segment X roughly quadrate with bulbous triangular dorsoapical lobes in dorsal view, with U-shaped excision in between. Apical margin of segment X in lateral view, with small, roughly lanceolate outgrowth medially, bearing few setae apically.

Inferior appendages longer than apex of segment X, each with clavate coxopodite in lateral view, bearing few elongate setae distally and shorter ones throughout. Coxopodite almost three times longer than harpago in lateral view; harpago flattened, almost equally wide throughout in lateral view and broader apically in ventral view.

Phallus in lateral view wider basally and apically, with somewhat rounded apex; in ventral view, with one pair of swellings and a very high, slightly narrowing apical part.

##### Remarks

Fore-wing length 8.2 mm, hind-wing length 6.1 mm in paratypes.

#### Diagnosis

Males of *Hydropsyche fitesa* sp. nov. are most similar to those of *H. tigrata*, but the following differences are noted: the subapical swellings of the phallus in *H. tigrata* are more pronounced in ventral and lateral views compared to *H. fitesa* sp. nov.; the apex of the phallus in lateral view is indented in *H. tigrata*, while it is rounded in *H. fitesa* sp. nov.; in *H. tigrata*, the dorsal margin of the phallus is apically depressed in lateral view, unlike in *H. fitesa* sp. nov., where it is nearly straight; the intersegmental profile between segments IX and X is shallower in lateral view in *H. tigrata*; in *H. tigrata*, the harpago is distally obliquely truncated in ventral view, resulting in a longer outer margin (in some Iranian specimens, the harpago is not extremely obliquely truncated distally, but it is still more dorsoapically produced), while, in *H. fitesa* sp. nov., the harpago is distally squarely truncated with similar lengths of the outer and inner margins; the dorsal corners of segment X are also longer and more pointed in *H. tigrata* in dorsal view. Furthermore, *Hydropsyche fitesa* sp. nov., like *H. sciligra*, differs from *H. tigrata* in the colouration of the fore-wings. *H. fitesa* sp. nov. has light brown fore-wings with irregularly speckled dark brown nuances throughout, while *H. tigrata* has light brown wings with three transverse bands.

Males of *Hydropsyche fitesa* sp. nov. also bear some resemblance to those of *H. iranica* and *H. sciligra* Malicky, but differ mainly in exhibiting a combination of the following characters: 1) in ventral view, only one pair of subapical swellings on the phallus, which are low and medium-wide, with margins above the swellings parallel; 2) phallus slightly curved around the basal third in lateral view; 3) dorsal keel of segment IX is wide, roughly rectangular, with a straight apical margin in dorsal view; 4) harpago is apically squarely truncated in ventral view; 5) intersegmental profile between segments IX and X is wide and deep in lateral view; 6) presence of a median outgrowth on the apical margin of segment X in lateral view. In *H. iranica*: 1) there is only one pair of subapical swellings onn the phallus, which are moderately high and narrow, with margins above the swelling narrowing significantly at the apex; 2) phallus is curved around the half of its length in lateral view; 3) dorsal keel of segment IX is roughly triangular in shape with a rounded apex in dorsal view; 4) harpago is apically rounded in ventral view; 5) intersegmental profile between segments IX and X is narrow and deep; 6) absence of an outgrowth on segment X in lateral view. In *H. sciligra*: 1) two pairs of swellings are present on the phallus, one pair located apically and the other subapically; 2) phallus is curved around the basal third in lateral view; 3) dorsal keel of segment IX is moderately wide and trapezoidal in dorsal view; 4) harpago is pointed ventroapically in lateral view; 5) intersegmental profile between segments IX and X is moderately wide and deep; 6) absence of an outgrowth on segment X in lateral view.

#### Etymology

The species is named after the first author’s wife, a lifelong supporter and participant in caddisfly studies across the world.

## Analysis

### Molecular analysis

The Bayesian Inference analysis revealed that all morphologically identified species formed monophyletic lineages with high nodal support values. The newly-described *Hydropsyche hindrajab* sp. nov. appeared as a sister lineage to *Hydropsyche sciligra*. These two lineages, together with *Hydropsyche* sp. (XJDQD332-18) from China and *Hydropsyche contubernalis* McLachlan, 1865, formed a well-supported clade that is sister to *Hydropsyche tigrata* and *Hydropsyche modesta* Navas, 1925 (Fig. 1). The Maximum Likelihood tree (Suppl. material 1) recovered similar species-level relationships, though with lower support values for deeper nodes. Both phylogenetic approaches consistently recovered *H. hindrajab* sp. nov. as a distinct, well-supported monophyletic lineage.

The three species delimitation methods (ASAP, ABGD and bPTP) showed high congruence in identifying species boundaries. All three approaches consistently recognised each morphologically defined species as a distinct molecular operational taxonomic unit (MOTU), including the newly-described *Hydropsyche hindrajab* sp. nov., *H. contubernalis*, *H. modesta* and *H. tigrata*, despite some variation in geographic origin amongst specimens. The undetermined *Hydropsyche* sp. (XJDQD332-18) from China was also recognised as a potentially distinct lineage by all three methods, warranting further taxonomic investigation.

Uncorrected pairwise genetic distances (*p*-distances) revealed clear differentiation amongst *Hydropsyche* species (Suppl. material 2). Intraspecific variation was consistently low, ranging from 0.2% (*H. hindrajab* sp. nov., *H. contubernalis* and *H. tigrata*) to 6.5% (*H. sciligra*), with *H. modesta* showing 0.5% divergence. The comparatively high intraspecific divergence observed in *H. sciligra* may indicate the presence of cryptic lineages within this species. In contrast, interspecific distances were substantially higher, ranging from 9.0% to 13.2%. The newly-described *H. hindrajab* sp. nov. exhibited minimal intraspecific divergence (0.2% between the two sequenced specimens), but showed substantial genetic divergence from its congeners. The closest relative, *H. sciligra*, differed by 10.0–10.2%, while distances to other species ranged from 10.8% (*Hydropsyche* sp. from China) to 12.8–12.9% (*H. modesta*).

## Discussion

**Figure 4.**
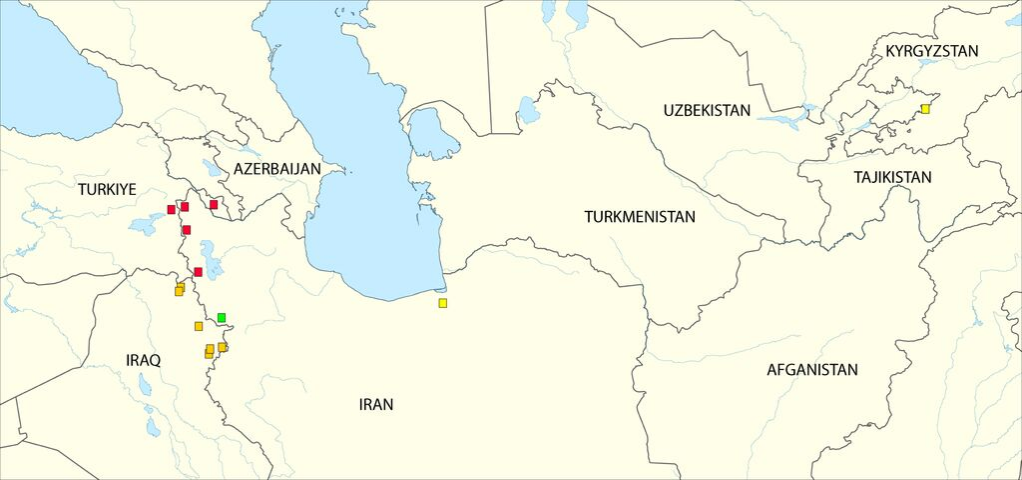
Distribution of *Hydropsyche hindrajab* sp. nov. (red squares), *Hydropsyche fitesa* sp. nov. (green square), *Hydropsyche sciligra* (yellow squares) and *Hydropsyche tigrata* (green squares), based on data used for the current study.

The discovery of two new Hydropsyche species from the Middle East and Caucasus ecoregions underscores the still insufficient level of faunistic investigation in these regions. While *Hydropsyche hindrajab* sp. nov. appears, based on current evidence, to be more widely distributed, *Hydropsyche fitesa* sp. nov. is presently known only from a single locality (Fig. 4). *Hydropsyche sciligra*, was originally described from the Demavend Mountains in Iran. Since its initial discovery it has been recorded in multiple locations across Iran, Türkiye and Azerbaijan. In his original description of this species, Malicky (1977) noted the presence of a single male specimen from the vicinity of Maku in West Azerbaijan Province, Iran which exhibited several morphological differences from typical *H. sciligra*, particularly the presence of pointed lobes on segment X, a square-shaped (rather than trapezoidal) dorsal keel on segment IX and parallel-sided apical margins of the phallus in lateral view. Malicky (1977) provisionally identified this specimen as *H. sciligra*, with the caveat that, if additional, similar specimens were discovered, it might warrant recognition as a distinct species. *Hydropsyche hindrajab* sp. nov. displays characters noted in this single specimen, although the apical part of the phallus, with its parallel margins, appears longer than depicted in Malicky’s illustrations (Malicky 1977, Malicky 2004).

*Hydropsyche tigrata*, initially described from Türkiye, has also been found in multiple localities in Iran. This species is readily distinguished by stable, prominent, large swellings on its phallus. On the other hand, *Hydropsyche iranica* is known from a limited number of localities in Iran, with its reported presence in Romania almost certainly a misidentification (Ciubuc 2016). As a member of the *Hydropsyche contubernalis* group, it is easily recognised by the narrowing apex of the phallus and the triangularly-shaped dorsal keel of segment IX.

The Bayesian Inference phylogenetic reconstruction recovered all morphologically defined species as distinct, well-supported monophyletic lineages (Fig. 1). This concordance at the species level was further corroborated by three independent molecular delimitation methods (ASAP, ABGD and bPTP). The agreement between morphological taxonomy and multiple lines of molecular evidence provides robust integrative support for the validity of these species, including the newly-described *H. hindrajab* sp. nov. While single-gene markers may have limited phylogenetic signal for resolving deeper relationships within rapidly diversifying lineages such as *Hydropsyche* (Rubinoff and Holland 2005), our integrative approach combining morphology, phylogenetics and multiple delimitation methods provides strong evidence for species-level distinctiveness.

Type localities of both new species in the West Azerbaijan Province of Iran are recognised for harbouring rare and unique species of aquatic insects (e.g. Ghaderi et al. (2024), Ibrahimi et al. (2024a)). This area likely harbours additional rare and undescribed species, thus contributing to the expanding knowledge of Iranian caddisflies (e.g. Ibrahimi et al. (2023a), Ibrahimi et al. (2023b), Ibrahimi et al. (2023c), Ibrahimi et al. (2024a), Ibrahimi et al. (2024b), Ibrahimi et al. (2025b), Oláh et al. (2025)). Twenty-three species of *Hydropsyche* are currently known from Iran, 67 from Türkiye and 12 from Azerbaijan and the potential for new species in these three countries belonging to the Middle East and Caucasus ecoregions remains high.

*Hydropsyche*, comprising more than 8% of all Trichoptera species recorded from the Western Palaearctic, is amongst the most diverse and ecologically important genera in this ecoregion (Ibrahimi 2024). Within this ecoregion, new species are continuously being described from areas such as North Africa and Balkans, including updates on range extensions and inventories from less investigated areas (e.g. Ibrahimi et al. (2019), Bilalli et al. (2024), Mabrouki et al. (2024), Samraoui et al. (2024), Bozdoğan et al. (2025), Ibrahimi et al. (2025a)) indicating that Western Palaearctic *Hydropsyche* remains incompletely known.

## Supplementary Material

XML Treatment for Hydropsyche hindrajab

XML Treatment for Hydropsyche fitesa

EF6CECB0-4339-5B7B-B173-0E807A2B33F3 10.3897/BDJ.14.e191076.suppl1 Supplementary material 1 Maximum Likelihood (ML) phylogeny based on *COI* sequences of *Hydropsyche* species Data type Image Brief description Maximum Likelihood (ML) phylogeny, based on *COI* sequences of *Hydropsyche* species, rooted with *Cheumatopsyche lepida*. Coloured bars on the right indicate species delimitation results from ASAP, ABGD and bPTP analyses. Node support values represent ML bootstrap support values. File: oo_1622854.png https://binary.pensoft.net/file/1622854 Halil Ibrahimi, Dora Hlebec

034F88A1-C2B2-56B2-BC9D-1E87900FA259 10.3897/BDJ.14.e191076.suppl2 Supplementary material 2 Range of uncorrected pairwise genetic distances (*p*-distances) Data type genomic Brief description Range of uncorrected pairwise genetic distances (*p*-distances) calculated from a 658-bp fragment of the *COI* gene. Gaps in the alignment were treated using pairwise deletion. File: oo_1599510.xlsx https://binary.pensoft.net/file/1599510 Halil Ibrahimi, Dora Hlebec

## Figures and Tables

**Table 1.** List of taxa and sequence data used in phylogenetic analyses. Newly-sequenced specimens are highlighted in bold.

**BOLD Process ID**	**Identification**	**Collection information**	**Latitude**	**Longitude**
**HYHIN001-26**	*Hydropsyche hindrajab* sp. nov.	Bardehsur River, Bardehsur Village, West Azerbaijan Province, Iran	37.437	44.827
**HYHIN002-26**	*Hydropsyche hindrajab* sp. nov.	Siah Cheshmeh, West Azerbaijan Province, Iran	39.060	44.295
IRCAD008-10	*Hydropsyche hindrajab* sp. nov.	Habash-e Sofla, Khoy, West Azerbaijan, Iran		
HMCAD0111-169	*Hydropsyche sciligra*	Kadamjay, 5 km NNW, Batken, Kyrgyzstan	40.166	71.7
HMCAD1211-18	*Hydropsyche sciligra*	Chesmeh, Dangan, 8 km S Ali, SE Elburs, Semnan, Iran	36	54
XJDQD332-18	*Hydropsyche* sp.	Yamadu, Ili, Zizhiqu, Xinjiang Uygur, China	43.767	81.952
IQCAD-0018	*Hydropsyche tigrata*	Chami Razan area, Peramagroon, Sulaymaniyah, Kurdistan, Iraq	35.805	44.976
IQCAD-0026	*Hydropsyche tigrata*	Kherazook, Peyran, Irbil, Kurdistan, Iraq	36.959	44.324
IQCAD-0044	*Hydropsyche tigrata*	Bekhma, Harier spelik, Irbil, Kurdistan, Iraq	36.709	44.277
IQCAD-0050	*Hydropsyche tigrata*	Banikani, Arabakan, Irbil, Kurdistan, Iraq	34.909	45.601
IQCAD-0060	*Hydropsyche tigrata*	Bani Khelan (Garmk), Sulaymaniyah, Kurdistan, Iraq	35.057	45.667
12IQTRA-0061	*Hydropsyche tigrata*	E Zalm village, Zalm stream, Kurmal, Halabja, Sulaymaniyah, Iraq	35.317	46.09
FBCAD023-10	*Hydropsyche contubernalis*	Altmuehl oberhalb Bruecke Groesdorf, 1.2 km N Kipfenberg, Bavaria, Germany	48.961	11.399
BCAI085-26	*Hydropsyche contubernalis*	Dyje, Pohansko, Chezs Republic	48.723	16.885
GRAEL7101-23	*Hydropsyche contubernalis*	Indre & Loire, Centre-Val de Loire, France	47.343	0.765
HMKKT224-10	*Hydropsyche modesta*	Pfaffstatten, Austria	48.03	16.25
AILG057-10	*Hydropsyche modesta*	near Pigadoulia, Greece	39.564	20.379
